# Sorafenib Plus Hepatic Arterial Infusion Chemotherapy in Advanced Hepatocellular Carcinoma: A Systematic Review and Meta-analysis

**DOI:** 10.5152/tjg.2023.22383

**Published:** 2023-04-01

**Authors:** Peichun Peng, Bin Wen, Ran Chen, Xin Deng

**Affiliations:** 1 International Zhuang Medicine Hospital Affiliated to Guangxi University of Chinese Medicine, Nanning, China; 2 Guangxi University of Chinese Medicine, Nanning, China; 3 Ruikang Clinical Faculty of Guangxi University of Chinese Medicine, Nanning, China; 4 Xiamen Humanity Hospital, Xiamen, China

**Keywords:** Sorafenib, hepatic arterial infusion chemotherapy, advanced hepatocellular carcinoma

## Abstract

This meta-analysis aimed to determine whether sorafenib combined with hepatic arterial infusion chemotherapy is beneficial for advanced hepatocellular carcinoma. We searched PubMed, Cochrane Library, and Embase to identify comparative studies evaluating sorafenib plus hepatic arterial infusion chemotherapy versus sorafenib for advanced hepatocellular carcinoma. Overall survival, progression-free survival, objective response rate, rate of progressive disease, and adverse events were evaluated. This meta-analysis included 5 randomized controlled trials (690 patients). Pooled estimates showed that compared with sorafenib, sorafenib plus hepatic arterial infusion chemotherapy was associated with higher overall survival (hazard ratio = 0.52, 95% CI: 0.28-0.95, *P* = .03), progression-free survival (hazard ratio = 0.57, 95% CI: 0.33-0.98, *P* = .04), objective response rate (risk ratio = 3.84, 95% CI: 1.23-12.05, *P* = .02), as well as higher rates of neutropenia (risk ratio = 7.90, 95% CI: 3.0-20.78, *P* < .0001) and thrombocytopenia (risk ratio = 2.73, 95% CI: 1.70-4.36, *P* < .0001), but had no significant influence for rate of progressive disease (risk ratio = 0.76, 95% CI: 0.43-1.37, *P* = .37). Sorafenib plus hepatic arterial infusion chemotherapy improved overall survival, progression-free survival, and objective response rate, but it had no effect on the rate of progressive disease. Combination therapy has a survival benefit for advanced hepatocellular carcinoma patients, and adverse events can be accepted. However, more large-scale randomized controlled trials are needed for further investigation.

**Main Points**

- Deng et al designed a meta-analysis to determine whether sorafenib combined with hepatic arterial infusion chemotherapy (HAIC) is beneficial for advanced hepatocellular carcinoma (HCC).
- The results showed that sorafenib plus HAIC improved overall survival, progression-free survival, and objective response rate, but it had no effect on the rate of progressive disease.
- Combination therapy has a survival benefit for advanced HCC patients, and adverse reactions can be accepted.
- However, more large-scale randomized controlled trials are needed for further investigation.

## INTRODUCTION

Hepatocellular carcinoma (HCC) is the third leading cause of malignancy-related deaths in the world^1,2^ and the second leading cause of death in China. However, due to the occult nature of this tumor, many HCC patients have intermediate or advanced disease at the time of diagnosis. Although surgical resection and local radiofrequency ablation are effective methods for the treatment of HCC, patients with intermediate-to-advanced disease have unfortunately missed the best time for these treatments.^3,4^ Furthermore, patients with advanced HCC cannot benefit from palliative therapy, and their median survival time is less than 1 year.^5^

Sorafenib is an oral multikinase inhibitor that inhibits several receptor tyrosine kinases and can inhibit tumor growth by influencing angiogenesis, tumor proliferation, and apoptosis.^6^ Since 2007, it has become the standard first-line treatment for advanced HCC. However, the outcomes of patients treated with sorafenib remain poor, with a median survival duration of only 5.5-7.2 months.^7,8^

Hepatic arterial infusion chemotherapy (HAIC), which is widely recommended in Asia, differs from systemic chemotherapy in that it delivers chemotherapeutic drugs directly to the tumor-supplying arteries, thereby minimizing systemic toxicity via hepatic metabolism. Studies have shown that for the management of advanced HCC associated with portal vein tumor thrombus, HAIC is better than sorafenib and might be a valuable treatment modality.^9,10^

In addition, studies have shown that sorafenib has synergistic effects with chemotherapeutic drugs,^11,12^ and some studies have suggested that sorafenib combined with HAIC might benefit patients with advanced HCC.^5,13^ However, other trials have indicated that survival time did not significantly differ between patients treated with sorafenib combined with HAIC and those treated with sorafenib alone.^14,15^ Therefore, there is insufficient evidence that sorafenib combined with HAIC improves survival in HCC. We designed the present meta-analysis to determine whether sorafenib combined with HAIC is beneficial for advanced HCC.

## MATERIALS AND METHODS

As the data for this study were obtained from published trials, there was no requirement for ethical approval or patient consent. The meta-analysis was conducted in accordance with the Preferred Reporting Items for Systematic Reviews and Meta-Analyses (PRISMA) statement.^16^

### Search Strategy

The PubMed, Cochrane library, and Embase databases were systematically searched for articles published up to February 1, 2022. The search terms included the following: “Sorafenib”[Mesh], “Carcinoma, Hepatocellular[Mesh],” “Liver Neoplasms[Mesh],” “Hepatic Arterial Infusion Chemotherapy,” “HAIC,” “group,” “trial.” To avoid the omission of useful articles, we manually searched the references cited in the relevant articles.

### Inclusion Criteria and Study Selection

The criteria used to screen the articles are listed as follows.

- Study design: randomized controlled trials (RCTs);
- Patients: advanced HCC patients, age ≥18 years;
- Intervention: sorafenib plus HAIC or sorafenib alone;
- Outcomes: provided at least one of the following outcomes of interest, namely, overall survival (OS), progression-free survival (PFS), rate of progressive disease (PD), objective response rate (ORR), and adverse events (AEs).

We excluded non-comparative studies, case reports, case series, retrospective studies, and studies with incomplete data. Two researchers were responsible for searching and screening the studies and extracting the data. If there were disagreements in the selection of articles, other members would be invited to participate in the discussion and reached an agreement.

### Data Extraction and Quality Assessment

The following data were extracted from each study: name of the first author, year of publication, study design, sample size, number of patients, intervention drugs, patient inclusion criteria, hazard ratios (HRs) with 95% CIs for OS and PFS, median OS and PFS, ORR, PD rate, and AEs. This step was performed by 2 researchers.

The quality of the RCTs was assessed using RevMan *v*5.3. We assessed the risk of bias for random sequence generation, allocation concealment, blinding of participants and personnel, blinding of outcome assessment, incomplete outcome data, selective reporting, and other bias.

### Statistical Analysis

We used Review Manager *version* 5.3 for statistical analysis. Two-tailed *P* <.05 were considered statistically significant. Pooled risk ratios (RR) with 95% CIs were calculated for the analysis of ORR, PD rate, and AEs. Pooled HRs with 95% CIs were calculated for the analysis of OS and PFS. The I^2^ statistic was used for the statistical assessment of heterogeneity among studies. If I^2^ <50%, we considered that there was no significant difference, and used the fixed-effects (FE) model to pool the study estimates. If I^2^ ≥50%, we considered that there was a significant difference and used the random-effects (RE) model to pool the study estimates. Sensitivity analysis was carried out by changing the effector model and statistical method.

## RESULTS

### Retrieved Literature

**Figure 1.**
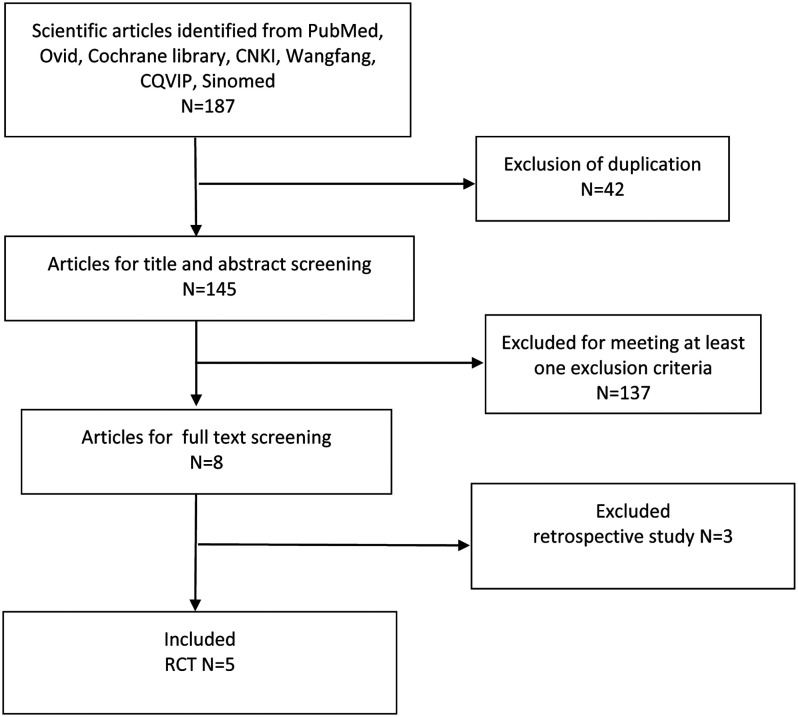
Flow chart of literature search and study-selection process.

**Figure 2.**
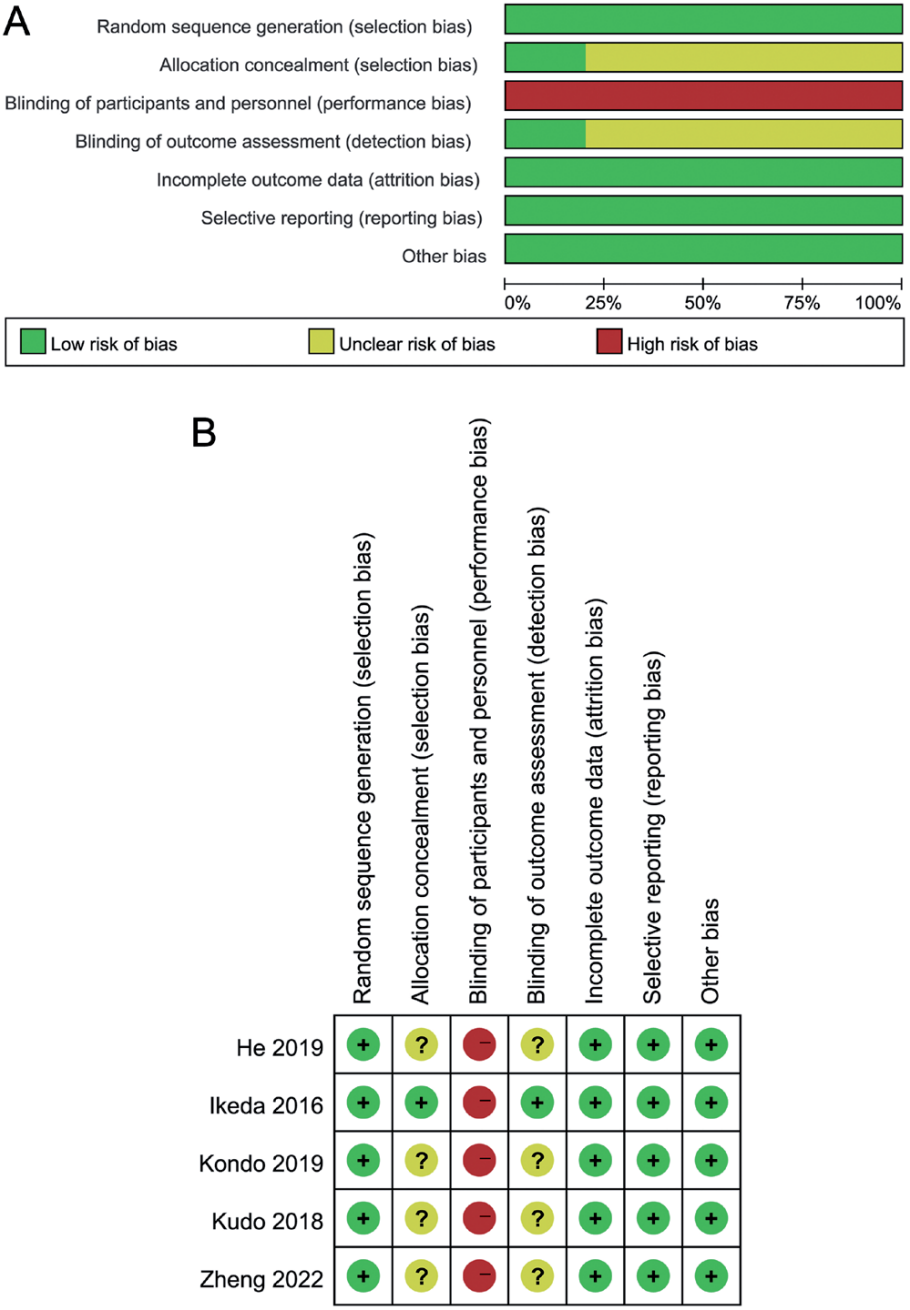
(A) The research quality of the RCTs, and (B) risk of bias graph risk of bias summary. RCTs, randomized controlled trials.

According to the search strategy, a total of 187 articles were retrieved, among which 42 were duplicated. After reading the titles and abstracts, we excluded a total of 137 references, and we excluded 3 studies after reading the full texts. Thus, finally, 5 RCTs were included in this meta-analysis (Figure 1). The research quality of the RCTs is shown in Figure 2.

### Study Characteristics

The 5 RCTs included in this meta-analysis were published between 2016 and 2022. The sample sizes of the individual studies varied between 64 and 247 subjects, with a total of 690 subjects. All studies included patients with vascular invasion and extrahepatic metastasis.^14,15,17-19^ Of the included patients, 337 received sorafenib plus HAIC (SoraHAIC group), while 331 received sorafenib. The protocol for HAIC was not consistent among the studies: cisplatin monotherapy was used in 2 studies,^14,18^ while the other 3 studies used multiple chemotherapeutic drugs.^15,17,19^ The main features of all the included studies are listed in Table 1.

### Overall Survival

**Figure 3.**
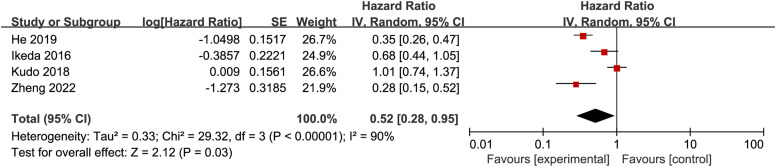
Forest plot comparing hazard ratios for overall survival between the SoraHAIC and sorafenib groups. SoraHAIC, sorafenib plus hepatic arterial infusion chemotherapy.

The HRs and median OS were shown in Table 2. Four studies reported the OS in the form of HRs.^15,17-19^ An random-effects (RE) model showed that SoraHAIC group had more advantage than the sorafenib group in OS (I^2^ = 90%, *P* < .00001; HR = 0.52, 95% CI: 0.28-0.95, *P* = .03) (Figure 3).

### Progression-Free Survival

**Figure 4.**
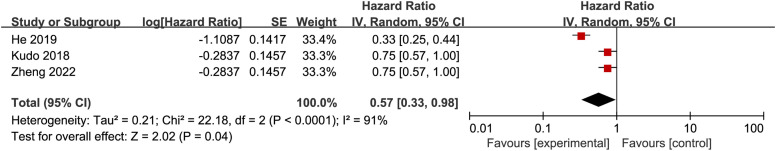
Forest plot comparing hazard ratio for progression-free survival between the SoraHAIC and sorafenib groups. SoraHAIC, sorafenib plus hepatic arterial infusion chemotherapy.

The HRs and median PFS were shown in Table 3. Three studies reported PFS in the form of HRs.^15,17,19^ An RE model showed significant difference between the SoraHAIC and sorafenib group (I^2^ = 91%, *P* < .0001; HR = 0.57, 95% CI: 0.33-0.98, *P* = .04) (Figure 4).

### Objective Response Rate and Rate of Progressive Disease

**Figure 5.**
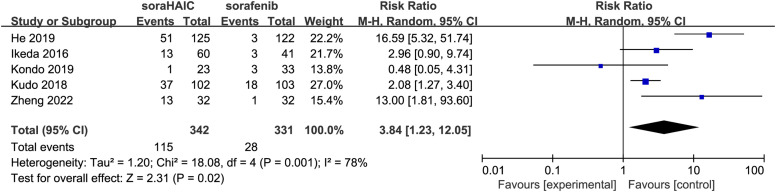
Forest plot comparing objective response rates between the SoraHAIC and sorafenib groups. SoraHAIC, sorafenib plus hepatic arterial infusion chemotherapy.

All studies reported the ORR.^14,15,17-19^ An RE model showed that the ORR was significantly higher in the SoraHAIC group than in the sorafenib group (I^2^ = 78, *P* = .001; RR = 3.84, 95% CI: 1.23-12.05; *P* = 0.02) (Figure 5, Table 4).

**Figure 6.**
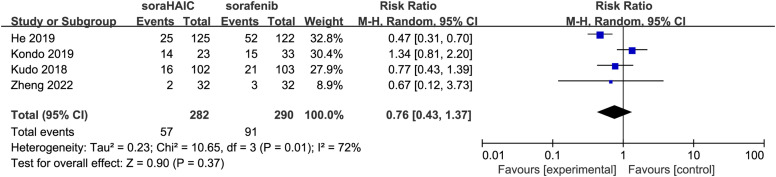
Forest plot comparing rate of progressive disease between the SoraHAIC and sorafenib groups. SoraHAIC, sorafenib plus hepatic arterial infusion chemotherapy.

All studies reported data on PD.^14,15,17-19^ An RE model showed that the rate of PD was not significant between the SoraHAIC group and sorafenib group (I^2^ = 72%, *P* = .01; RR = 0.76, 95% CI: 0.43-1.37, *P* = .37) (Figure 6, Table 4).

### Adverse Effects

**Figure 7.**
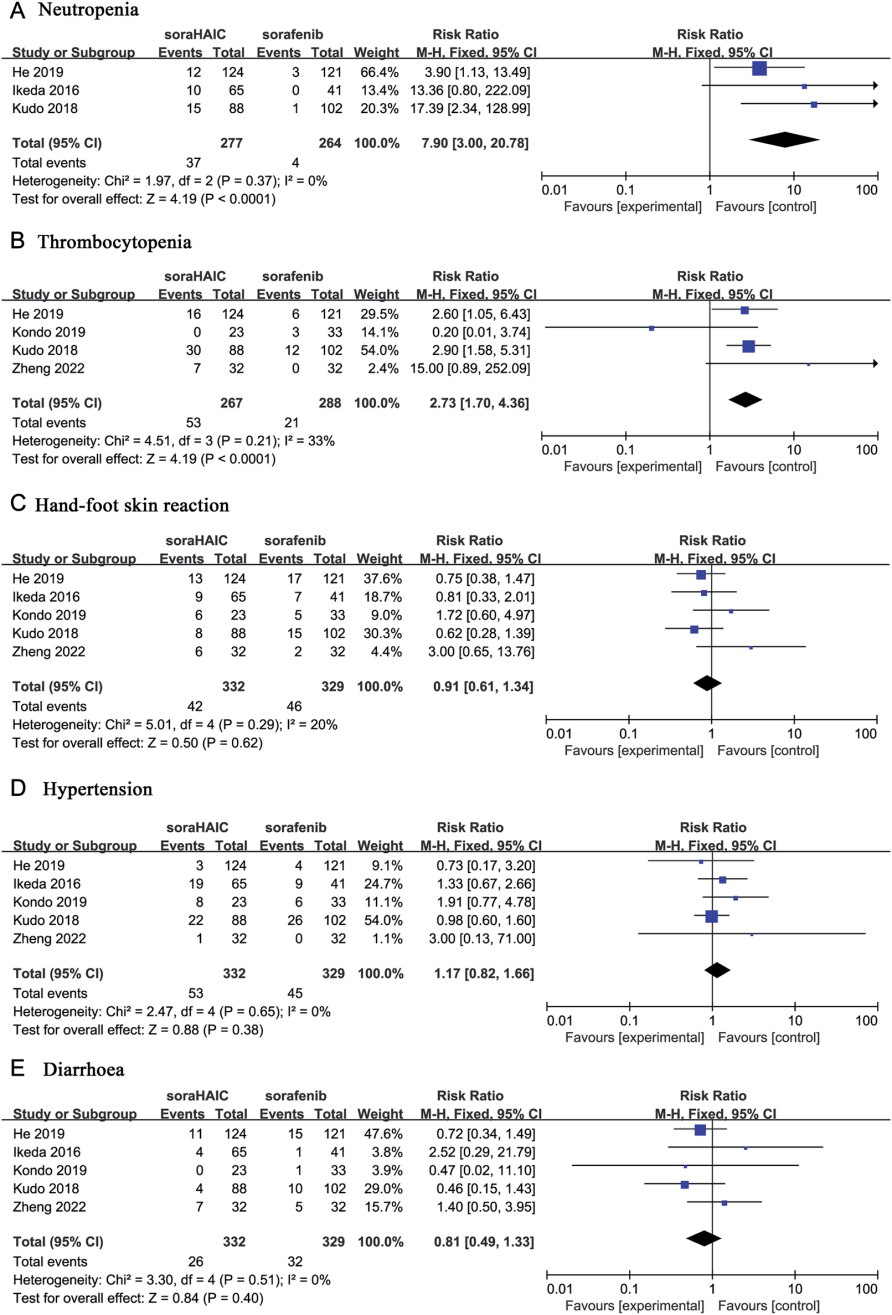
Forest plots comparing adverse effects between the SoraHAIC and sorafenib groups. (A) Neutropenia; (B) thrombocytopenia; (C) hand–foot syndrome; (D) hypertension; (E) diarrhea. SoraHAIC, sorafenib plus hepatic arterial infusion chemotherapy.

Adverse effects were reported in 5 studies.^14,15,17-19^ The most common AEs were neutropenia, hand–foot syndrome, thrombocytopenia, hypertension, and diarrhea (Figure 7A to 7E). Compared to the sorafenib group, the SoraHAIC group showed significantly higher rates of neutropenia^15,17,18^ (I^2^ = 0, *P* = .37; RR = 7.90, 95% CI: 3.0-20.78, *P* < .0001)(Figure 7A) and thrombocytopenia^14,15,17,19^ (I^2^ = 33%, *P* = .21; RR = 2.73, 95% CI: 1.70-4.36, *P* < .0001) (Figure 7B).

## DISCUSSION

The median survival time of patients with advanced HCC is only 1-2 years.^20^ In recent years, studies have suggested that the combination therapy with PD-L1 (atezolizumab) and vascular endothelial growth factor (VEGF) (bevacizumab) will bring in the very next future in patients with advanced HCC.^21,22^ However, because of the tumor heterogeneity, multidisciplinary treatments, including combination or sequential treatments, might be beneficial to advanced HCC patients. Sorafenib is an oral drug that inhibits multiple tyrosine kinases and exhibits anti-proliferative and anti-angiogenic effects. Since 2007, it has been a first-line therapy for unresectable HCC.^23^ A systematic review which included 7 RCTs showed that sorafenib could significantly improve OS (HR = 50.74, 95% CI: 0.61-0.90, *P* = .002) and time to progression (TTP) (HR = 50.69, 95% CI: 0.55-0.86, *P* = .001) in patients with advanced HCC.^24^ However, some studies have reported a low response rate to sorafenib, with no complete responses and few partial responses.^25,26^ Furthermore, drug-related AEs reportedly necessitated permanent drug discontinuation in 11% of patients, dose interruptions in 44% of patients, and dose reductions in 26% of patients.^27^ These reasons led to limitations in the use of sorafenib.

Compared with other therapies, HAIC has been proven to prolong median survival time and improve response rates in patients with advanced HCC.^28,29^ One study compared the efficacy of HAIC (using low-dose 5-fluorouracil and cisplatin) with sorafenib for advanced HCC.^30^ The results showed that the median survival time and the median time to treatment failure were significantly longer in the HAIC group than in the sorafenib group; thus, the authors concluded that HAIC was preferred for the treatment of HCC associated with Vp3/Vp4 tumor thrombus.^30^ Another study reported that compared with sorafenib, HAIC treatment significantly prolonged OS and TTP. These results suggested that HAIC was a valuable therapeutic modality for advanced HCC associated with portal vein tumor thrombus.^8^ In addition, a meta-analysis indicated that compared with sorafenib, HAIC has significant advantages in terms of ORR and disease-control rate, and concluded that HAIC may replace sorafenib for the treatment of advanced HCC.^10^ These benefits are likely attributable to the fact that HAIC delivers drugs directly to the tumor-supplying blood vessels through the hepatic artery, thereby increasing the intrahepatic drug concentration and reducing systemic toxicity.^31,32^ Saeki et al^33^ found that for advanced HCC patients with significant vascular invasion and Child-Pugh class A or class B disease, the first­line treatment should be HAIC. In Japan, HAIC is recommended as a treatment option for unresectable HCC.^34,35^ A phase I/II study showed that the oral administration of sorafenib combined with the intra-arterial infusion of low-dose cisplatin plus 5-fluorouracil was a promising treatment for advanced HCC patients.^36^ Another study suggested that sorafenib sensitizes human HCC cells to cisplatin via suppression of Wnt/β-catenin signaling.^11^ Therefore, some researchers^17^ considered that combination treatment with HAIC and sorafenib might have complementary effects in advanced HCC.

The present meta-analysis results suggested that sorafenib combined with HAIC significantly improved survival benefits for advanced HCC patients. Moreover, combination therapy was also associated with a significant improvement in the overall response and progression-free survival. However, we found that sorafenib plus HAIC did not have a significant effect on terms of PD. Moreover, our study showed that most of the common AEs occurred at a comparable rate between the SoraHAIC and sorafenib groups. While neutropenia and thrombocytopenia were significantly more frequent in the SoraHAIC group, fortunately, the AEs were not severe and were manageable by treatment interruption or dose modification. In addition, He et al^17^ reported that during HAIC treatment, the microcatheter position needed to be changed to prevent collateral arterial re-embolization.

In brief, the combination of HAIC and sorafenib is likely to have a survival benefit for patients with advanced HCC. Compared with systemic chemotherapy, HAIC has a stronger anti-tumor effect and a lower incidence of side effects. In addition, sorafenib can effectively reduce the tumor burden and have a synergistic effect with chemotherapy drugs, so the combined efficacy is better than sorafenib alone. Whereas the adverse effects of the combination therapy are not serious and can be alleviated by adjusting the dose or discontinuing the treatment, hence, the risk is still bearable.

The above results provide us with some inspiration for surgical treatment for patients with advanced HCC. While surgical resection and liver transplantation (LT) are still the optimal therapy for patients with early-stage HCC, LT remains the best treatment strategy as it not only replaces the diseased liver but also restores normal liver function.^37,38^ However, there are strict selection and allocation criteria for LT, including attention to tumor biology, tumor size and number, leading to the exclusion of many HCC patients from the list of LT. Downstaging may give advanced HCC patients the chance to receive LT.^39^ Our meta-analysis shows that sorafenib plus HAIC can improve OS, PFS, and ORR in advanced HCC patients. These results indicate that combination therapy may provide a direction for the treatment of tumor degradation before LT. A study backs up our judgment; it reported a patient with advanced HCC beyond the Milan criteria. He successfully reduced the stage of HCC after adjuvant therapy with sorafenib plus HAIC and underwent LT.^40^ In addition, the large number of patients and organ shortage led patients who were already on the waitlist for LT to develop disease progression during the long donor waiting period and be excluded from the transplant criteria. Bridging therapy may reduce the risk of dropout due to tumor progression.^41,42^ While our study showed that sorafenib plus HAIC cannot improve the rate of PD, which suggested combination therapy is not helpful for bridging therapy, it is regrettable that there is lack of clinical evidence to support our conjecture.

Some inevitable deficiencies of our meta-analysis must be acknowledged. First, few studies were included, and the sample size was small; one study^15^ conducted the statistical analysis without providing the predetermined sample size, which might have skewed the results. When reporting OS, another study^14^ included 12 patients who did not receive subsequent sorafenib treatment; therefore, this study was excluded from the analysis of OS. Thus, the sample sizes might have affected the reliability of the results. Second, moderate or substantial heterogeneity was observed in the pooled analyses of OS, PFS, ORR, and PD rate, which might be due to confounders in the included studies. This suggests that these studies may not be suitable for merged analysis. The source of heterogeneity was identified as being possibly related to study design, patient characteristics, chemotherapeutic drugs and doses, and comparators. Third, a study was inconsistent with the other four in combination therapy procedures, this may be one of the main sources of heterogeneity. We considered that heterogeneity was the main factor affecting the reliability of the results. Besides, all the studies on HAIC come from the Asian-Pacific world and have not been replicated in the Western world, so further studies are needed to determine whether combination therapy is beneficial to patients in Western countries.

## CONCLUSION

In summary, the present meta-analysis evaluated the safety and efficacy of combined sorafenib plus HAIC for advanced HCC patients, showing that combination therapy leads to a survival benefit for advanced HCC patients. It can not only improve the objective response rate of patients, but also prolong OS, and adverse reactions can be accepted. The results provide us with some inspiration for LT for patients with advanced HCC. However, these results may not be reliable enough to guide clinical decision-making, and more large-scale RCTs are needed for further investigation.

## Figures and Tables

**Table 1.** Baseline Characteristics of Included Studies in the Meta-analysis

Study	Study Design	Random Method	Inclusion Criteria	Treatment and Patients	HAIC Protocols	Child-Pugh (A/B/C)	ECOG PS(0-1/≥2)	Macroscopic Vascular Invasion (Yes/No)	Extrahepatic Spread (Yes/No)
He et al^17^	RCT	1:1, computer-generated randomization	Age ≥18 years; hepatocellular carcinoma (not suitable for curative surgery, or local ablation); patients had portal vein invasion confirmed by 2 imaging techniques; Child-Pugh A class liver function, an Eastern Cooperative Oncology Group performance status of 0 to 2; no previous treatment for hepatocellular carcinoma, at least one measurable lesion according to Response Evaluation Criteria in Solid Tumors (RECIST) version 1.1; adequate bone marrow, liver, and renal function.	Sorafenib (122) vs. SoraHAIC (125)	Oxaliplatin + fluorouracil + leucovorin	247/0/0	183/64	247/0	80/167
Ikeda et al^18^	RCT	1:1.6, Randomization was done centrally using a minimization method withbiased-coin assignment	Age 20-79 years old; advanced HCC; unsuitable for surgical resection, liver transplantation, local ablative therapy or transarterial chemoembolization (TACE); no prior history of chemotherapy; Child-Pugh score 5-7; adequate bone marrow, liver, and renal function.	Sorafenib (41) vs. SoraHAIC (65)	Cisplatin	96/10/0	106/0	57/49	32/74
Kondo et al^14^	RCT	1:1, Random number was generated by SAS 9.3	Age ≥20 years; patients who were unlikely to benefit from surgical resection or locoregional treatment, had a life expectancy of 12 weeks or more; Eastern Cooperative Oncology Group (ECOG) performance status score of 0 or 1; Child-Pugh score of seven or less; Previous treatment was terminated at least 4 weeks before this study entry.	Sorafenib (33) vs. SoraHAIC (35)	Cisplatin	60/8/0	NR	43/25	18/50
Kudo et al^15^	RCT	1:1, Randomization was done centrally via an interactive web response system	Age ≥20 years; advanced hepatocellular carcinoma not suitable for resection, local ablation, or transarterial chemoembolization; ECOG performance status 0-1; Child-Pugh score seven or lower; adequate bone marrow, liver, and renal function.	Sorafenib (103) vs. SoraHAIC (102)	Cisplatin+fluorouracil	182/22/1	205/0	122/83	53/152
Zheng et al^19^	RCT	1:1, Randomization was conducted using arandom number table	Age 18-75 years old; inoperable advancedprimary HCC with major PVTT, including tumor thrombosisin the main trunk (Vp4) and first branch (Vp3) of the portalvein; histologic or clinical diagnosis of HCC (23); at least one measurable lesion; Child-Pugh class A disease; no history of treatment; Eastern Cooperative Oncology Group performance status of 0-2; life expectancy of 2 months or more; and adequate organ function	Sorafenib (32) vs. SoraHAIC (32)	Oxaliplatin+5-fluorouracil+leucovorin	55/9/0	59/5	9/55	9/55

HAIC, hepatic arterial infusion chemotherapy; NR, not reported; RCT, randomized controlled trials; SoraHAIC, sorafenib plus HAIC.

**Table 2.** The Median OS and HR in 2 Groups

Study	SoraHAIC Group (95% CI) (Months)	Sorafenib Group (95% CI) (Months)	HR (95% CI)	*P*
He et al^17^	13.37 (10.27-16.46)	7.13 (6.28-7.98)	0.35 (0.26-0.48)	<.001
Ikeda et al^18^	10.6 (NR)	8.7 (NR)	0.68 (0.44-1.049)	.073
Kondo et al^14^	N/A	N/A	N/A	N/A
Kudo et al^15^	11.8 (9.1-14.5)	11.5 (8.2-14.8)	1.009 (0.743-1.371)	.955
Zheng et al^19^	16.3(0-35.5)	6.5 (4.4-8.6)	0.28 (0.15-0.53)	<.001

HAIC, hepatic arterial infusion chemotherapy; HR, hazard ratio; NA, not available; NR, not reported; OS, overall survival; SoraHAIC, sorafenib plus HAIC.

**Table 3.** The Median PFS and HR in Two Groups

Study	SoraHAIC Group (95% CI) (Months)	Sorafenib Group (95% CI) (months)	HR (95% CI)	*P*
He et al^17^	7.03 (6.05-8.02)	2.6 (2.15-3.05)	0.33 (0.25-0.43)	<.001
Ikeda et al^18^	NR	NR	NR	NR
Kondo et al^14^	NR	NR	NR	NR
Kudo et al^15^	4.8 (3.4-6.2)	3.5 (2.6-4.4)	0.753 (0.566-1.003)	.051
Zheng et al^19^	9.0 (4.4-13.6)	2.5 (1.3-3.7)	0.26 (0.15-0.47)	<.001

HAIC, hepatic arterial infusion chemotherapy; HR, hazard ratio; NR, not reported; PFS, progression-free survival; SoraHAIC, sorafenib plus HAIC.

**Table 4.** The ORR and PD in Two Groups

Study	Outcome (ORR/PD)	SoraHAIC Group (Event/Total)	Sorafenib Group (Event/Total)
He et al^17^	ORR	51/125	3/122
	PD	25/125	52/122
Ikeda et al^18^	ORR	13/60	3/41
	PD	NR	NR
Kondo et al^14^	ORR	1/23	3/33
	PD	14/23	15/33
Kudo et al^15^	ORR	37/102	18/103
	PD	16/102	21/103
Zheng et al^19^	ORR	13/32	1/102
	PD	2/32	13/32

HAIC, hepatic arterial infusion chemotherapy; NR, not reported; ORR, objective response rate; PD, of progressive disease; SoraHAIC, sorafenib plus HAIC.
